# Cost comparison by treatment arm and center‐level variations in cost and inpatient days on the phase III high‐risk B acute lymphoblastic leukemia trial AALL0232

**DOI:** 10.1002/cam4.1206

**Published:** 2017-12-23

**Authors:** Amanda M. DiNofia, Alix E. Seif, Meenakshi Devidas, Yimei Li, Matthew Hall, Yuan‐Shung V. Huang, Viviane Cahen, Stephen P. Hunger, Naomi J. Winick, William L. Carroll, Brian T. Fisher, Eric C. Larsen, Richard Aplenc

**Affiliations:** ^1^ Division of Oncology The Children's Hospital of Philadelphia Philadelphia Pennsylvania; ^2^ Department of Pediatrics University of Pennsylvania Perelman School of Medicine Philadelphia Pennsylvania; ^3^ Division of Biostatistics University of Florida Gainesville Florida; ^4^ The Center for Clinical Epidemiology and Biostatistics University of Pennsylvania Perelman School of Medicine Philadelphia Pennsylvania; ^5^ Children's Hospital Association Lenexa Kansas; ^6^ Center for Pediatric Clinical Effectiveness The Children's Hospital of Philadelphia Philadelphia Pennsylvania; ^7^ Division of Hematology‐Oncology Department of Pediatrics Center for Cancer and Blood Disorders University of Texas at Southwestern Medical Center Dallas Texas; ^8^ Perlmutter Cancer Center Departments of Pediatrics and Pathology NYU Langone Medical Center New York New York; ^9^ Division of Infectious Diseases The Children's Hospital of Philadelphia Philadelphia Pennsylvania; ^10^ Division of Pediatric Hematology/Oncology Maine Medical Center Scarborough Maine

**Keywords:** Child, clinical trial, costs, leukemia, variation

## Abstract

The Children's Oncology Group (COG) develops and implements multi‐institutional clinical trials with the primary goal of assessing the efficacy and safety profile of treatment regimens for various pediatric cancers. However, the monetary costs of treatment regimens are not measured. AALL0232 was a COG randomized phase III trial for children with acute lymphoblastic leukemia that found that dexamethasone (DEX) was a more effective glucocorticoid than prednisone (PRED) in patients younger than 10 years, but PRED was equally effective and less toxic in older patients. In addition, high‐dose methotrexate (HD‐MTX) led to better survival than escalating doses of methotrexate (C‐MTX). Cost data from the Pediatric Health Information System database were merged with clinical data from the COG AALL0232 trial. Total and component costs were compared between treatment arms and across hospitals. Inpatient costs were higher in the HD‐MTX and DEX arms when compared to the C‐MTX and PRED arms at the end of therapy. There was no difference in cost between these arms at last follow‐up. Considerable variation in total costs existed across centers to deliver the same therapy that was driven by differences in inpatient days and pharmacy costs. The more effective regimens were found to be more expensive during therapy but were ultimately cost‐neutral in longer term follow‐up. The variations in cost across centers suggest an opportunity to standardize resource utilization for patients receiving similar therapies, which could translate into reduced healthcare expenditures.

## Introduction

The adult healthcare community has embraced health economics as a necessary tool in maximizing the value of medical care, which is the optimal patient outcome at the lowest cost 1. While the pediatric literature has been slower to adopt cost research, pediatric healthcare providers have increasingly become interested in the quality and costs of care delivery. Though pediatric research in this area lags significantly behind the adult literature, a growing body of economics research does exist in pediatric oncology.

Few studies identifying pediatric cancer costs exist in bone marrow transplant, acute lymphoblastic leukemia (ALL), and rhabdomyosarcoma 2, 3, 4, 5, 6. There are data, although not in pediatric cancer, demonstrating variations in costs among hospitals delivering similar care 7, 8, 9. However, no study has compared costs of care for patients randomized to different treatment arms of a large ALL clinical trial or compared costs of care for patients receiving the same on‐study therapy but at different centers.

The Children's Oncology Group (COG) is a cooperative group that designs and implements multi‐institutional clinical trials. Most children with newly diagnosed cancer in the United States (US) are enrolled in COG clinical trials 10. COG has not collected data that would allow for cost analyses despite the importance of cost on the delivery of pediatric cancer therapy 1. Our research group has successfully merged data from COG with administrative data from the Pediatric Health Information System (PHIS; Children's Hospital Association, Lenexa, KS) and illustrated the ability to leverage these data to estimate inpatient costs on a COG acute myeloid leukemia trial 11. This prior work has established the foundation for comparative cost analyses between treatment arms of a trial.

AALL0232 was a COG phase III pediatric B‐ALL trial that randomized patients upfront to 14 days of dexamethasone (DEX) or 28 days of prednisone (PRED) during an Induction phase and to high‐dose methotrexate with leucovorin rescue (HD‐MTX) or escalating “Capizzi” methotrexate plus pegaspargase (C‐MTX) in the Interim Maintenance I (IM 1) phase. The trial found that HD‐MTX had superior event‐free survival (EFS) when compared to C‐MTX, and, for patients <10 years of age, DEX was superior to PRED 12. The determination of superior treatment regimens establishes one component of value‐based care analyses. By merging AALL0232 trial data with PHIS cost data, we aimed to assess the second component of value‐based care analyses in comparing the costs between treatment arms and across centers. It was hypothesized that PHIS inpatient costs would be greater for the HD‐MTX and DEX arms, room and board and pharmacy costs would be the largest drivers of total cost, and costs would vary across centers to deliver the same therapy.

## Methods

### Study population

The study population included patients enrolled on COG AALL0232 who were successfully linked to the PHIS dataset. AALL0232 enrolled patients between the ages of 1 and 30 years with high‐risk B‐ALL from December 29, 2003 until January 21, 2011 12. Patients enrolled on AALL0232 but not randomized were excluded from the cohort. Patients without inpatient data during the COG treatment period, enrolled at hospitals that do not report cost data, or missing individual cost data were also excluded.

### Merged AALL0232 and PHIS database

Patients enrolled on AALL0232 at a PHIS contributing center were linked to their PHIS data using common elements in the two data sources 13. A successful match required the following data elements to be the same in the COG and PHIS datasets: hospital, date of birth, date of diagnosis, and gender. Only patients who matched on all criteria were included in the cohort.

The final dataset was inclusive of data elements that were unique to the COG and PHIS data sources. COG provided data specific to a patient's trial participation: date of enrollment, treatment arm, end of Induction response, date of relapse, and date of last follow‐up or end of study date. COG data also informed demographic and insurance variables. PHIS data were inclusive of information generated from billing charges specific to care provided in the inpatient setting, emergency department, and observation unit at 44 free‐standing US pediatric hospitals 13. Clinic data were not comprehensively captured in PHIS within the timeframe that AALL0232 was open to enrollment; thus, this information was not available for this study. PHIS data informed assessment of inpatient care costs as described further below.

### PHIS adjusted inpatient costs

PHIS provides daily inpatient adjusted charges for each patient for the following components: pharmacy; laboratory services; clinical, which is comprised mostly of surgical and procedural costs; supplies, which includes medical devices and surgical equipment; imaging, and room and board. Daily adjusted inpatient cost was calculated by multiplying PHIS Health Care Financing Administration wage/price index adjusted charges by the appropriate ratio of cost to charge (RCC) provided by each hospital and standardized to the 2013 consumer price value 11. A gold standard for cost calculation in pediatric epidemiologic research does not exist; therefore, there is no way to validate these cost measurements. However, other research groups have used PHIS adjusted costs in their economic analyses 14. The derived daily component costs were summed for each patient resulting in total cost. For patients missing component cost data, total adjusted inpatient cost provided by PHIS was used to derive average daily cost.

### Covariates

The relationship of treatment arm and cost with age (≥1 year to <5 years, ≥5 years to <10 years, ≥10 years to <15 years, and ≥15 years), gender, race (White, Black, American Indian or Alaskan Native, Asian, Native Hawaiian or other Pacific Islander, or other), and insurance status (public, private, or other) was explored.

### Statistical analysis

All statistical analyses were performed using STATA (version 13, StataCorp, College Station, TX) and Prism (version 7, GraphPad, La Jolla, CA). Statistical significance was determined by a two‐sided *P* < 0.05. Demographic variables for patients enrolled on AALL0232 and successfully merged to PHIS data were compared to patients on AALL0232 but not merged to ensure the cohort was representative of the trial participants. Medians with interquartile ranges (IQR) were calculated for age; frequencies with percentages were calculated for gender, race, and geographic region. To compare the two groups, a Wilcoxon rank sum test was used for age and a chi‐square test was used for gender, race, and geographic region.

Standard descriptive statistics (medians, IQR) were summarized for total costs per randomized arm of all patients in the cohort at the following time intervals: at the completion of the courses of therapy involved in the randomization, which was IM 1 for the methotrexate arms and Induction for the steroid arms, at the completion of protocol therapy, and at the time of last follow‐up. Component costs were compared between randomized arms at these same timeframes to identify drivers of total cost. Daily inpatient costs were compared between the randomized arms for the courses involved in the randomization. Standard descriptive statistics and a Wilcoxon rank sum test were used to compare length of follow‐up at the end of protocol and the last follow‐up timeframes. A Wilcoxon rank sum test was used to perform the univariate analysis of adjusted inpatient total costs and component costs by randomized arm at each time interval and daily costs during the randomized courses only. A linear regression model was used to compare costs between randomized arms, adjusting for the covariates above. As cost was not normally distributed, it was log transformed prior to inclusion in the linear regression model. Patients were censored at relapse in all timeframe analyses except the last follow‐up timeframe; therefore, the last follow‐up analysis includes costs associated with salvage therapy. In each timeframe, patients were censored at time of death and when removed from study protocol.

Variation in total cost across hospitals was investigated for patients randomized to HD‐MTX during IM 1. Median and IQR for total costs were tabulated for each hospital with patients on the HD‐MTX arm during IM 1. A Kruskal‐Wallis test was used to determine whether the total costs were significantly different across hospitals.

It is possible that patients who suffer a significant toxicity from therapy may incur increased costs that could skew the estimates for a specific hospital. To establish a more uniform comparison between hospitals, a second analysis comparing total cost, pharmacy cost, and hospital days including only patients who received HD‐MTX according to the proposed course schedule was performed. Patients were deemed to have received HD‐MTX according to schedule if they had pharmacy billing codes for four doses of HD‐MTX within the proposed 67‐day course. Patients who received four doses of HD‐MTX within 67 days were compared to patients who did not receive four doses in that timeframe using a Wilcoxon rank sum test for age and a Chi‐square test for gender and race.

### Sensitivity analyses

Two sensitivity analyses were performed. The first analysis aimed to estimate how much less the C‐MTX costs would need to be in comparison to HD‐MTX for the difference in cost to remain statistically significant. Since C‐MTX is administered in the outpatient setting at most institutions, we are missing the costs of C‐MTX administration for patients randomized to this arm. We sequentially inputted additional costs to the C‐MTX arm during IM 1 to represent the missing outpatient administration costs and compared these arbitrary totals using a Wilcoxon rank sum test to the existing HD‐MTX data.

The second analysis was performed to account for patients who may have received additional, unplanned cycles of chemotherapy that would increase cost totals. Patients randomized to C‐MTX at the time the AALL0232 interim analysis was reported on April 1, 2011 were rerouted to a HD‐MTX arm if they were still on therapy and had not yet completed the first cycle of Maintenance. The first cycle of Maintenance for slow early responders could occur as late as 14 months after the start of therapy. Therefore, patients enrolled prior to February 2010 could not be re‐routed. Analysis of total cost was performed on only those patients enrolled before February 2010 to see if cost estimates changed.

## Results

**Figure 1:**
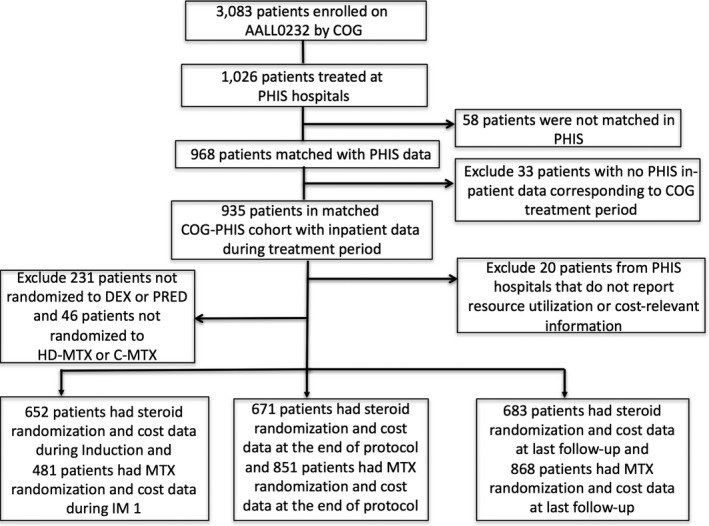
Consort Diagram for COG AALL0232‐PHIS Merged Cohort. Only patients randomized to DEX versus PRED during Induction or HD‐MTX versus C‐MTX during IM 1 were included in the cost comparisons between arms. COG, Children's Oncology Group; PHIS, Pediatric Health Information System; DEX, dexamethasone; PRED, prednisone; HD‐MTX, high‐dose methotrexate; C‐MTX, Capizzi methotrexate; IM 1, Interim Maintenance.

Of the 3,083 patients COG enrolled on AALL0232, 935 patients matched within the COG and PHIS databases and had inpatient data within the COG treatment period, which is 91% of the patients enrolled by COG and treated at PHIS institutions and 30% of the total patients enrolled by COG (Fig. 1). The merged patients were not significantly different in gender or race from the unmerged patients. While there was a statistically significant difference in age between the groups, this difference did not appear to be clinically significant. Merge status did vary statistically significantly by region as well. Only hospitals within the United States (US) are included in the PHIS database; therefore, patients enrolled and cared for outside of the US are not eligible for the merge. While international patients were excluded from the comparison, merge status was compared among the Northeast, Midwest, South, and West regions of the US. The Northeast region varied the most dramatically; the unmerged group had twice as many patients as the merged group (20.4% vs 10.1%). When this region is excluded from the analysis, the regions no longer differ significantly by merge status. There are a smaller number of PHIS hospitals in the Northeast relative to the population and hospital density in that region. Therefore, an increased number of patients in this region likely receive oncology care in non‐PHIS institutions compared to other US regions.

### Total costs by course

There was a significant difference in total cost between the DEX and PRED arms during Induction in both the univariate and multivariable analyses (*P* = 0.047 and 0.040, respectively) with a median total inpatient cost for the DEX arm being $37,724 (IQR: $21,449–66,650) and median total inpatient cost for the PRED arm being $33,554 (IQR: $20,793–55,738; Table 1). Despite DEX having a greater median total inpatient cost when compared to PRED during Induction, PRED had a greater median daily inpatient cost at $2915 (IQR: $2301–3682) when compared to DEX at $2,663 (IQR: $2227–3453) that was significant (*P* = 0.026).

**Table 1:** Total cost comparison by randomized arm

Arm	*N*	Cost, median (IQR)	Unadjusted *P* value	Adjusted *P* value
Induction				
DEX	322	$37,724 (21,449–66,650)	0.047	0.040
PRED	330	$33,554 (20,793–55,738)		
IM 1				
HD‐MTX	346	$38,891 (30,800–53,795)	<0.001	<0.001
C‐MTX	135	$15,786 (6,307–37,802)		
End of protocol				
DEX	329	$107,125 (58,949–168,724)	0.013	0.068
PRED	342	$89,172 (49,402–142,772)		
HD‐MTX	445	$109,296 (61,229–164,374)	<0.001	<0.001
C‐MTX	406	$78,738 (38,512–147,692)		
Last follow‐up				
DEX	338	$155,242 (86,918–327,544)	0.990	0.947
PRED	345	$152,366 (83,143–374,166)		
HD‐MTX	453	$160,107 (104,207–319,356)	0.329	0.188
C‐MTX	415	$157,305 (74,826–411,838)		

DEX, dexamethasone; PRED, prednisone; IM 1, Interim Maintenance 1; HD‐MTX, high‐dose methotrexate; C‐MTX, Capizzi methotrexate.

During IM 1, the median total inpatient cost of HD‐MTX was significantly more at $38,891 (IQR: $30,800–53,795) than the median cost of C‐MTX at $15,786 (IQR: $6307–37,802) in both the univariate and multivariable analyses (*P* < 0.001; Table 1). However, there was no difference in median daily inpatient cost during IM 1 between the methotrexate arms [median cost $2134 (IQR: $1678–2620) versus $2068 (IQR: $1651–2727) for HD‐MTX versus C‐MTX arm, *P* = 0.818].

### Total costs at the end of protocol therapy and at last follow‐up

There was not a significant difference in follow‐up inpatient days between the randomized arms at the completion of protocol therapy or at last follow‐up (Table S1). There was a significant difference in median total inpatient costs at the end of protocol therapy between DEX and PRED arms in the univariate analysis and near significant difference in the multivariable analysis [median cost $107,125 (IQR: $58,949–168,724) versus $89,172 (IQR: $49,402–142,772) for DEX versus PRED, unadjusted *P* = 0.013, adjusted *P* = 0.068; Table 1]. There was also a significant difference in median total inpatient costs between HD‐MTX and C‐MTX. The median total inpatient cost for HD‐MTX was $109,296 (IQR: $61,229–164,374) versus $78,738 (IQR: $38,512–147,692) for C‐MTX (*P* < 0.001; Table 1).

While there was a significant difference in median total inpatient costs for the duration of protocol therapy between DEX and PRED, there was not a significant difference in cost between these arms at time of last follow‐up: median cost $155,242 (IQR: $86,918–327,544) versus $152,366 (IQR: $83,143–374,166) for DEX versus PRED, unadjusted *P* = 0.990, adjusted *P* = 0.947 (Table 1). Similarly, the difference in median total costs between the HD‐MTX and C‐MTX arms present for the on‐protocol therapy timeframe did not persist at last follow‐up [median cost $160,107 (IQR: $104,207–319,356) versus $157,305 (IQR: $74,826–411,838) for HD‐MTX versus C‐MTX, unadjusted *P* = 0.329, adjusted *P* = 0.188; Table 1].

### Component costs

During Induction, the median inpatient costs for all components of care were greater on the DEX arm when compared to the PRED arm. This difference was significant in both the univariate and multivariable analyses for supply and room and board costs, univariate analysis only for imaging costs, and multivariable analysis only for the laboratory costs. The median pharmacy, supply, laboratory, imaging, and room and board costs for the DEX arm were significantly greater than the PRED arm during protocol therapy in both the univariate and multivariable analyses. Similar to the total cost analysis, there was no difference in component costs between the DEX and PRED arms at last follow‐up in the univariate or multivariable analyses.

All of the cost components had significantly greater median inpatient costs on the HD‐MTX arm when compared to the C‐MTX arm during IM 1 in both the univariate and multivariable analyses, except for imaging (Table 2). Unadjusted imaging costs associated with the C‐MTX arm were significantly greater than the HD‐MTX arm. However, the costs were very small for imaging, and the adjusted difference was not statistically significant. All cost components were significantly greater in the HD‐MTX arm for the on‐protocol timeframe when compared to the C‐MTX arm except for supply in the univariate analysis and imaging in both the univariate and multivariable analyses. There was no significant difference in component costs between HD‐MTX and C‐MTX at time of last follow‐up.

**Table 2:** Cost driver comparison between HD‐MTX and C‐MTX arms for IM 1

Cost Driver	Arm	Cost, median (IQR)	Unadjusted *P* value	Adjusted *P* value
Pharmacy	HD‐MTX	$8,111 (5,132–12,975)	<0.001	<0.001
	C‐MTX	$3,233 (890–8021)		
Supply	HD‐MTX	$410 (116–1243)	<0.001	<0.001
	C‐MTX	$140 (19–489)		
Lab	HD‐MTX	$2608 (1890–4017)	<0.001	<0.001
	C‐MTX	$1511 (480–3620)		
Imaging	HD‐MTX	$0 (0–279)	0.001	0.940
	C‐MTX	$75 (0–622)		
Clinical	HD‐MTX	$1724 (215–4746)	<0.001	0.006
	C‐MTX	$607 (0–2295)		
Room & Board	HD‐MTX	$22,226 (17,268–30,583)	<0.001	<0.001
	C‐MTX	$9437 (3662–23,202)		

IM 1, Interim Maintenance 1; HD‐MTX, high‐dose methotrexate; C‐MTX, Capizzi methotrexate.

### Variation across PHIS hospitals

**Figure 2:**
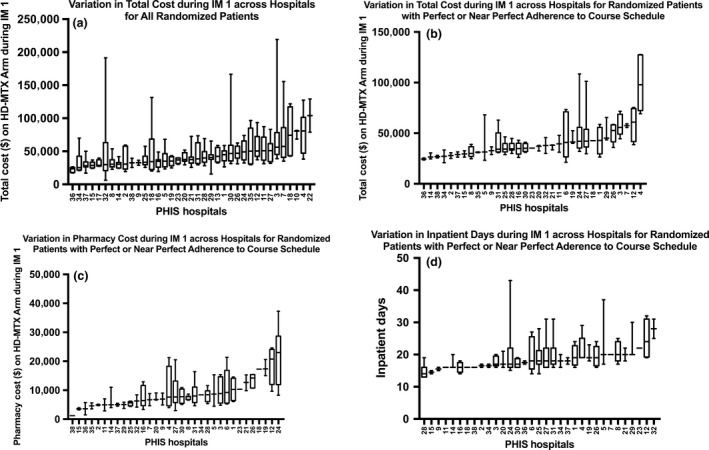
Boxplots of variation across hospitals during IM 1. (A) Total costs for all patients. (B) Total costs for all patients who adhered to the course schedule. (C) Pharmacy costs for all patients who adhered to the course schedule. (D) Inpatient days for all patients who adhered to the course schedule. Edge of box represents interquartile range, line in center of box represents median, edge of vertical lines extending from box represents 10th and 90th percentiles. *X*‐axis on all figures labelled with mock numbers representing PHIS hospitals.

For patients on the HD‐MTX arm, total median costs during IM 1 ranged from $23,524 (IQR: $17,954–25,515) to $103,953 (IQR: $78,857–129,050) (Fig. 2A). This variation in cost across hospitals was significant (*P* < 0.001). No significant difference in age or race existed between the patients who adhered to the proposed IM 1 schedule compared with those who did not (Table 3). More females than males did not receive four doses of HD‐MTX in the 67‐day proposed timeframe. After excluding patients who did not receive HD‐MTX according to the proposed course, the total median costs were still significantly different across hospitals (*P* < 0.001). Total median costs during IM 1 ranged from $24,520 (IQR: $23,524–25,515) to $97,919 (IQR: $72,407–127,342) (Fig. 2B). Pharmacy costs and inpatient days during IM 1 also remained significantly different across hospitals (*P* < 0.001 and *P* = 0.005, respectively). Pharmacy median costs ranged from $1,188 in a hospital with only one patient in this category to $23,023 (IQR: $11,950–28,680) (Fig. 2C). Hospital days during IM 1 ranged from 14 days (IQR: 13–16 days) to 28 days (IQR: 25–31 days) (Fig. 2D).

**Table 3:** Comparison of patients with good schedule adherence versus patients with poor schedule adherence to IM 1

	Good IM 1 adherence(*n* = 124)	Poor IM 1 adherence (*n* = 222)	*P* value
Age median, (IQR)	11.4 (4.1–15.5)	11.8 (4.2–15.1)	0.985
Female *N*, (%)	45 (36.3%)	112 (50.5%)	0.011
Race *N*, (%)			
Black	9 (7.3%)	15 (6.8%)	0.585
White	86 (69.4%)	165 (74.3%)	
Other	29 (23.4%)	42 (18.9%)	

IM 1, Interim Maintenance 1.

### Sensitivity analyses

The first sensitivity analysis was performed to elucidate how much more C‐MTX would have to cost to lose the statistically significant difference between total costs of the methotrexate arms during IM 1. Outpatient costs between $18,000 and $19,000 would have to be missing from the C‐MTX arm during IM 1 to lose the statistically significant difference found between the methotrexate arms at this interval (Table S2).

The second sensitivity analysis revealed that 43 patients in the merged cohort were randomized to C‐MTX within 14 months of the interim analysis, which made them eligible to be re‐routed to a HD‐MTX course. Excluding these patients resulted in similar results to the primary analysis characterized by higher median total inpatient cost for HD MTX ($163,105, IQR: $102,942–326,970) when compared to C‐MTX ($151,802, IQR: $70,332–425,386). No statistically significant difference in total costs existed between these groups (*P* = 0.232) at last follow‐up.

## Discussion

Although pediatric cancer diagnoses are not common, healthcare costs associated with these diseases are substantial. Investigating ways to maximize treatment outcomes has long been a goal of pediatric oncologic research, and now interest in learning ways to minimize the costs of care delivery is growing 15, 16. Accurate measures of pediatric oncologic costs are under‐represented in the literature, and costs of administration of upfront therapy for B ALL have not been published. Understanding the value, which includes both quality and cost, of pediatric oncology care is critical for all stakeholders to determine the ideal therapeutic approach. While we were not able to completely characterize value in ALL care, we did identify accurate inpatient costs to administer the standard of care therapy for the most common pediatric malignancy. Even in the absence of addressing quality, these cost estimates are novel and get us closer to addressing value in this population.

The cost comparison between DEX and PRED revealed that total inpatient costs for DEX were significantly greater in the adjusted analysis than for PRED during the induction period, and this difference approached significance for the entire protocol therapy timeframe. The etiology for this increased cost is not known. Assessing specific components of the overall costs provides some insights to possible sources for the difference. Supply costs, which include orthopedic devices and surgical equipment, clinical costs, which include surgical and procedural bills, and imaging costs were higher in the DEX arms during the on‐protocol period. We hypothesize that these cost component differences could reflect the increased osteonecrosis events reported for patients randomized to the DEX arm 17. Querying PHIS data for osteonecrosis diagnoses or specific surgical or pain medicine billing codes was beyond the scope of this paper but would be a potential area for future investigation.

Despite DEX having greater total inpatient costs during Induction, PRED had significantly higher daily costs during that period. Since our analysis on the components of total cost showed that room and board was the greatest driver of total cost, we investigated whether a difference in length of inpatient stay would explain this discrepancy. The median inpatient days during Induction for DEX were 14 (IQR: 8–24) and were 12 (IQR: 6–21) for PRED. This difference was statistically significant (*P* = 0.002). Since the total costs were within a $5000 range, the longer length of stay (LOS) on the DEX arm largely explains the lower daily costs despite the higher total costs.

Patients receiving HD‐MTX incurred significantly more inpatient costs than patients on the C‐MTX arm during IM1 and throughout the protocol period. At almost all institutions, HD‐MTX was administered in the inpatient setting, and C‐MTX was given in the outpatient clinic. As we are not capturing outpatient data, it is not surprising that C‐MTX had lower associated costs. The sensitivity analysis revealed that $18,000 to $19,000 would need to be missing in outpatient C‐MTX costs for the HD‐MTX and C‐MTX cost comparison to lose significance. The primary analysis demonstrated that room and board was the greatest cost driver with a median total cost on the HD‐MTX arm during IM 1 of $22,226. It is reasonable to conclude that the missing outpatient costs on the C‐MTX arm do not total the $19,000 necessary to lose statistical significance, because it can be assumed that costs associated with an inpatient stay are greater than $3000 more than outpatient administration costs. Therefore, even though we are missing outpatient administration costs on the C‐MTX arm, we are confident that the significant difference in costs between these arms during IM 1 would persist even if these outpatient costs were able to be included. While the sensitivity analysis addresses the missing outpatient costs, the best approach to this limitation of our dataset would be to input outpatient data from another source, which will be conducted in a future analysis.

In investigating the drivers of total cost, room, and board was found to be the greatest contributor to total cost by a considerable margin. Pharmaceutical costs were the next largest driver of cost after inpatient hospital stay. While we did investigate the drivers of cost associated with this ALL therapy, we did not develop a predictive model for cost. To perform predictive modeling, preliminary work must exist to inform variables chosen for the models. No data currently exists in the literature on the cost of upfront ALL therapy; therefore, this data serves a basis to perform predictive modeling in the future.

The data specific to inpatient expenditures during IM 1 supports that substantial variation exists across institutions when caring for children receiving the same chemotherapy regimen. Significant differences were demonstrated even in the analysis that only included patients who received HD‐MTX similar to the proposed course schedule. This sub‐analysis should have eliminated patients who suffered toxicity that extended their therapeutic course, resulting in increased costs and prolonged hospital stays. Quality data were not available in the retrospective dataset; however, HD‐MTX, as the arm with the greatest EFS, can service as a proxy for the quality component of value. The variation in adjusted costs to deliver HD‐MTX across hospitals demonstrates that this identical “highest quality” therapy, even in the setting of clinical trials, is administered at a wide variety of costs. Therefore, the greatest quality care can still be administered at a cost saving.

Bessaha et al. identified predictors of the length and cost of inpatient stays for adult patients with psychotic disorders using an inpatient database of US community hospitals 18. The drivers of the identified variations in cost and inpatient stay from our study are not known. It is possible that there are differences in methotrexate level thresholds for discharge or variations in costs of supportive medications used. Identifying predictors that could explain the cost differential across hospitals by performing a factor or cluster analysis would be a potential future area of study, now that we have definitively shown that significant cost variation exists. Regardless, these data suggest that the opportunity to reduce healthcare costs may be through standardizing the utilization of resources beyond the actual chemotherapy regimens.

The primary limitation of this study is the lack of outpatient cost within the PHIS data. This limitation did not impact the cost comparison between DEX versus PRED arms, but the comparison of HD‐MTX versus C‐MTX was vulnerable to the lack of outpatient data, as most patients received C‐MTX as an outpatient. Future comparisons of therapeutic arms that vary in location of care need to consider both inpatient and outpatient costs. Another limitation to the study is generalizability. Patients managed at non‐PHIS sites are not included in the study. Only 30% of patients enrolled on AALL0232 were captured in the COG and PHIS merged cohort. However, the assembled cohort was shown to be similar to the unmerged AALL0232 patients (Table 4).

**Table 4:** Comparison of merged cohort versus unmerged study patients

	Merged patients (*n* = 935)	Unmerged patients (*n* = 2148)	*P* value
Age median, (IQR)	11.7 (5.2–14.9)	12.3 (5.8–15.5)	0.005
Female *N*, (%)	418 (44.7%)	957 (44.6%)	0.937
Race *N*, (%)			
Black	67 (7.2%)	149 (6.9%)	0.945
White	700 (74.9%)	1620 (75.4%)	
Other	168 (18.0%)	379 (17.7%)	
Region *N*, (%)			
Northeast	94 (10.1%)	363 (20.4%)	<0.001
Midwest	252 (27.0%)	432 (24.3%)	
South	311 (33.3%)	561 (31.5%)	
West	278 (29.7%)	423 (23.8%)	
Outside US	0	369	

This study is the first to evaluate costs of the treatment of pediatric ALL in the US. Cost data from an administrative dataset can augment the clinical data already collected by the COG and allow accurate estimates of costs of care delivery. The information learned from these merged datasets can serve as a foundation for building value‐based models for care delivery. While the clinically superior arm of HD‐MTX was more expensive during therapy, costs were equivalent between HD‐MTX and C‐MTX at last follow‐up. This convergence of cost likely represents costs associated with salvage therapy in the less efficacious arm; this assumption should be evaluated in future studies. These data support that the most efficacious upfront therapy, even if it is more expensive, is cost‐neutral in the long‐term. The considerable hospital‐level variation in costs and inpatient days to deliver the same therapy found in this study demonstrates that HD‐MTX can be administered in a more cost‐effective manner. Therefore, cost savings can still be achieved without sacrificing survival.

## Conflict of Interest

None declared.

## Supporting information


**Table S1.** Total inpatient follow‐up days by randomized arm.
**Table S2.** Inputting arbitrary costs on C‐MTX arm during IM 1 to determine magnitude of cost difference necessary to maintain HD‐MTX's statistically significant higher costs
