# Supplementary material for: Cost comparison by treatment arm and center‐level variations in cost and inpatient days on the phase III high‐risk B acute lymphoblastic leukemia trial AALL0232
Source: Cancer Med. 2017 Dec 23;7(1):3–12. doi: 10.1002/cam4.1206 (PMC5773964; doi:10.1002/cam4.1206)
Supplement: Supplementary file 1 — Table S1. Total inpatient follow‐up days by randomized arm. Table S2. Inputting arbitrary costs on C‐MTX arm during IM 1 to determine magnitude of cost difference necessary to maintain HD‐MTX's statistically significant higher costs [file CAM4-7-3-s001.docx]

Supplementary Table 1. Total Inpatient Follow-Up Days by Randomized Arm

|  | Inpatient Days,  Median (IQR) | p value |
| --- | --- | --- |
| End of Protocol | | |
| DEX | 441 (160-841) | 0.813 |
| PRED | 424 (151-837) |  |
| HD-MTX | 386 (152-830) | 0.979 |
| C-MTX | 413 (106-836) |  |
| Last Follow-Up | | |
| DEX | 689 (278-1068) | 0.106 |
| PRED | 742 (309-1178) |  |
| HD-MTX | 672 (276-1083) | 0.239 |
| C-MTX | 701 (312-1120) |  |
| DEX, dexamethasone; PRED, prednisone; HD-MTX, high-dose methotrexate; C-MTX, Capizzi methotrexate | | |

Supplementary Table 2. Inputting Arbitrary Costs on C-MTX Arm During IM 1 to Determine Magnitude of Cost Difference Necessary to Maintain HD-MTX’s Statistically Significant Higher Costs

| HD-MTX Median Cost | New C-MTX Median Cost | p value |
| --- | --- | --- |
| $38,891 | *+ $10,000 =*  $25,786 | <0.001 |
| $38,891 | *+ $15,000 =*  $30,786 | <0.001 |
| $38,891 | *+ $18,000 =*  $33,786 | 0.019 |
| $38,891 | *+ $19,000 =*  $34,786 | 0.071 |
| $38,891 | *+ $20,000 =*  $35,786 | 0.213 |
| IM 1, Interim Maintenance 1; HD-MTX, high-dose methotrexate; C-MTX, Capizzi methotrexate | | |
